# Calcium Phosphate Cement with Antimicrobial Properties and Radiopacity as an Endodontic Material

**DOI:** 10.3390/ma10111256

**Published:** 2017-10-31

**Authors:** Tzong-Ming Shieh, Shih-Ming Hsu, Kai-Chi Chang, Wen-Cheng Chen, Dan-Jae Lin

**Affiliations:** 1 Department of Dental Hygiene, China Medical University, Taichung 404, Taiwan; tmshieh@mail.cmu.edu.tw (T.-M.S.); wencchen@fcu.edu.tw (W.-C.C.); 2 School of Dentistry, College of Medicine, China Medical University, Taichung 404, Taiwan; 3 Department of Biomedical Imaging and Radiological Sciences, National Yang Ming University, Taipei 112, Taiwan; smhsu@ym.edu.tw; 4 Advanced Medical Devices and Composites Laboratory, Feng Chia University, Taichung 407, Taiwan; chernlin2@gmail.com; 5 Department of Fiber and Composite Materials, Feng Chia University, Taichung 407, Taiwan

**Keywords:** calcium phosphate cement, hinokitiol, tricalcium silicates, endodontic materials, antibacterial

## Abstract

Calcium phosphate cements (CPCs) have several advantages for use as endodontic materials, and such advantages include ease of use, biocompatibility, potential hydroxyapatite-forming ability, and bond creation between the dentin and appropriate filling materials. However, unlike tricalcium silicate (CS)-based materials, CPCs do not have antibacterial properties. The present study doped a nonwashable CPC with 0.25–1.0 wt % hinokitiol and added 0, 5, and 10 wt % CS. The CPCs with 0.25–0.5 wt % hinokitiol showed appreciable antimicrobial properties without alterations in their working or setting times, mechanical properties, or cytocompatibility. Addition of CS slightly retarded the apatite formation of CPC and the working and setting time was obviously reduced. Moreover, addition of CS dramatically increased the compressive strength of CPC. Doping CS with 5 wt % ZnO provided additional antibacterial effects to the present CPC system. CS and hinokitiol exerted a synergic antibacterial effect, and the CPC with 0.25 wt % hinokitiol and 10 wt % CS (doped with 5 wt % ZnO) had higher antibacterial properties than that of pure CS. The addition of 10 wt % bismuth subgallate doubled the CPC radiopacity. The results demonstrate that hinokitiol and CS can improve the antibacterial properties of CPCs, and they can thus be considered for endodontic applications.

## 1. Introduction

Calcium phosphate cements (CPCs) are biocompatible materials that possess a self-hardening feature, also referred to as self-setting bone cements, and have been used in medical and dental applications for many years. CPCs can be formed by mixing one or more types of calcium phosphate salts with a liquid. The liquid can be distilled water, phosphoric acid, or some other alkaline water. Most final products of the CPC reaction are hydroxyapatites (HA), dicalcium phosphate dihydrate, and amorphous calcium phosphate (ACP). When in contact with body fluids, ACP precipitates form a more stable phase, HA. CPCs are advantageous for use in hard or connective tissue repair because of their inherent chemical affinity, nonexothermic properties, injectability, and paste-like consistency, which allows them to fit into different types of bone voids and contour defects. Recently, many researchers have attempted to improve CPC components for shortening the setting time and achieving an adjustable resorption time [1,2,3,4]. Furthermore, infection control using antibacterial bone grafts or substitutes has heralded a new era of CPC development. CPCs can be endowed with antibacterial properties by using two modification methods: The first method entails producing Ca(OH)_2_ during the reaction process [5], and the second method involves using hardened CPC as a drug delivery carrier to release antibacterial agents. The second method can be further divided into two types based on different antibacterial agents: The first type involves incorporating antibacterial ions in the hardening reaction, for example, alkali ion-substituted [6], silver ion-doped [7], and bismuth ion-doped CPCs [8]; and the second type entails incorporating antibiotics that do not alter the setting time [9,10,11].

CPCs with antimicrobial properties have the necessary attributes for endodontic materials. CPCs combined with tricalcium silicate (Ca_3_SiO_5_, CS) were considered as dental endodontic materials [12]. CS is the main component of mineral trioxide aggregates (MTAs), which are popular dental endodontic materials (used for root canal filling, root-end filling, root or furcal perforation repair, open apices, and direct pulp capping) [13,14]. However, MTAs have drawbacks including poor handling [15], high solubility [16,17], and a relatively long setting time [18,19]. Therefore, the use of highly biocompatible biomaterials, such as CPCs modified with small amounts of hinokitiol, as endodontic filling materials may be of great interest.

Hinokitiol is a natural component isolated from *Chamacyparis taiwanensis*, and it exhibits antibacterial, antifungal, antiviral, antiplasmodial, and insecticidal activities [20,21] without developmental toxicity or carcinogenicity. Hinokitiol inhibits oral bacteria but exhibits low cytotoxicity to normal oral cells, and it has been used in toothpastes, oral care gels, and root canal sealers to improve the oral lichen planus, reduce halitosis, and enhance the antibacterial activity of sealers [22]. The antimicrobial and odontogenesis properties were reported to be enhanced in hinokitiol-containing calcium silicate cements [23]. Furthermore, hinokitiol was determined to inhibit inflammatory responses mediated by nuclear factor kappa B, hypoxia-inducible factor-1α (HIF-1α), prostaglandins, and tumor necrosis factor-α [24].

Because of its high antimicrobial activity and low cytotoxicity, hinokitiol has received considerable attention in research on dental antibacterial materials recently. Our previous study demonstrated that an appreciable proportion of hinokitiol can improve the antimicrobial activity of root canal sealers, inhibit the mRNA expression of inflammation genes such as cyclooxygenase-2 and HIF-1α in MG-63 cells and human gingival fibroblasts (HGFs), and downregulate the mRNA levels of lysyl oxidase in HGFs [22]. Hinokitiol was added to β-Ca_2_SiO_4_ cement for application as a dental root canal filling. Huang et al. [23] developed a cement with antibacterial properties that could also increase the bioactivity of odontoblasts.

In this study, we used a newly developed CPC as the substrate, which possesses an antiwash feature that could be advantageous for sealing [25], and supplemented it with different weight ratios of hinokitiol, CS, or hinokitiol/CS. This study evaluated the antibacterial properties, cytotoxicity, phase structures, and microstructures of the hinokitiol-containing CPCs; the synchronized antibacterial effects of hinokitiol and CS were also examined, as well as the radiopacity of CPCs after the addition of bismuth subgallate (C_6_H_2_(OH)_3_COOBi(OH)_2_, BS) as a radiopacifier [26].

## 2. Materials and Methods

### 2.1. CPC Sample Preparation and Characterization

A nondispersive original CPC powder was prepared from tetracalcium phosphate (TTCP)/dicalcium phosphate anhydrous (DCPA) as described previously [27,28]. The CS samples were fabricated in our lab as described previously [29,30], and CS/Z powder was obtained by modifying in-house CS with 5 wt % ZnO. In brief, CS/Z was prepared by sintering the mixed powders of CaCO_3_, SiO_2_, and ZnO at the respective weight ratios of 79.8, 16.0, and 4.2 wt %. The powders were dispersed in ethanol (30 g in 100 mL) and stirred at room temperature for 1 day and dried. After the raw powders were completely mixed and dried, they were heated up to 900 and 1400 °C at heating rates of 6 and 3.3 °C/min, respectively. The powders were calcined for 5 h, followed by direct quenching. After cooling, the powders were ground and sieved through a 325-mesh sieve (0.044 mm) and characterized. To form a mixture slurry, the weighed powders were premixed through ball milling for 1 h. The hinokitiol powder (Sigma-Aldrich, St. Louis, MO, USA) was weighed (0.001–0.008 g) and added to 0.8 g of the CPC powder. The final powders were mixed with 0.28 mL of hardening solution [1 M (NH_4_)_2_HPO_4_] at pH 8.1. The pastes were filled in a stainless steel mold to form cylinder specimens of 12 mm height and 6 mm diameter for the compressive strength test, and disc specimens of 3 mm thickness and 6 mm diameter were prepared for all other tests. After 15 min, the samples were pushed out and placed under saturated vapor pressure at 37 °C for 24 h prior to tests. The methods used for measuring setting and working times and compressive strength were the same as those described in a previous study [26]. In brief, the compressive test was conducted in accordance with ASTM F 451-99a by using a desktop universal mechanical tester (LS 500, LLOYD Instruments, Tokyo, Japan) at a crosshead speed of 2 mm/min. The setting and working times were determined using a Vicat needle (400 g, 1 mm diameter).

### 2.2. Morphological Observations and Structural Analysis

A field-emission scanning electron microscope (SEM, JSM-6700F JEOL, Tokyo, Japan) was used to investigate the morphology of the prepared cements. An X-ray diffraction (XRD) apparatus (D8 Discover SSS BRUKER, Karisruhe, Germany) with Cu-Kα radiation (λ = 0.154 nm) at 40 kV and 40 mA was used to identify the various cement phases. The XRD tests were conducted using a step scan, in which the angle (2θ) was set between 20° and 35° with a step size of 0.02°. The phase structures were identified by comparing the peaks with the data from the Joint Committee on Powder Diffraction Standards-Powder Diffraction File.

### 2.3. Microorganism Culture and Chemicals

*Staphylococcus aureus* (ATCC number: 25923) and *Escherichia coli* (ATCC number: 10798) were used in this study. *S. aureus* and *E. coli* cells were cultured in Luria–Bertini broth. Each culture was inoculated by loop transfer from frozen tube slants containing 3 mL of nutrient broth agar. The cells were cultured at 37 °C for 24 h, followed by transfer onto an appropriate solid medium and overnight incubation. Selected colonies were transferred to an appropriate liquid medium and incubated for 4–6 h to achieve log-phase growth. The optical density at 600 nm (OD600) of each culture was adjusted to 1.0 using fresh broth to obtain a standard inoculum of 10^6^ colony-forming units (CFU)/mL, which was verified by counting colony numbers on agar media following a 10-fold series dilution. Stock cultures were maintained at −80 °C in growth broth containing 15% sterile glycerol.

### 2.4. Inhibition Zones (Agar Diffusion Test)

Liquid 1.5% agar broth was equilibrated in a 50 °C water bath for 30 min after autoclave sterilization. Cultures were inoculated with 10^6^ CFU/mL glycerol stock by swirling, followed by pouring them into 10-cm plates. Three setting cement specimens were placed on the surface of the solidified tryptic soy broth (TSB) agar, and the cultures were incubated for 24 h at 37 °C. The diameter of the inhibition zone was recorded and photographed [9].

### 2.5. Quantitative Evaluation of Antibacterial Properties

After the samples were sterilized by autoclaving, they were placed in 2 mL of TSB culture solution to achieve an OD595 of 0.2 to 1 × 10^7^ cells/mL. The conductivity changes with bacterial growth were monitored during incubation at 37 °C for 1, 4, 8 h, 1 day, and 2 days. The amount of bacteria was quantitated by resuspending in 100 μL of culture medium and recording OD595 values using a microplate reader. Each assay was repeated three times. To eliminate the artifacts of color changes due to bismuth ion release, the quantitative evaluation of BS-containing samples was performed by retransferring the conditioned media onto an appropriate solid medium, followed by overnight incubation. The Fourier transform infrared (IR) spectra were measured on a spectrophotometer (Spectrum TWO, PerkinElmer, Waltham, MA, USA) and transmission spectra of the samples were obtained by forming a thin transparent KBr pellet (1:100 *w*/*w*).

### 2.6. Cytocompatibility

The cytotoxicity test was performed using the murine fibroblast cell line L929 in accordance with ISO 10993-5. The L929 cells were grown in alpha-minimum essential medium (α-MEM, Gibco, Gaithersburg, MD, USA) containing nonessential amino acids and supplemented with 10% horse bovine serum, 100 mg/mL of streptomycin, and 100 U/mL penicillin. The L929 cells were maintained in a humidified atmosphere at 5% CO_2_ and 37 °C. The culture medium was renewed two times a week. The water-soluble tetrazolium salt (WST) assay was performed to evaluate cell proliferation at 1 day after the initial seeding of 5 × 10^4^ L929 cells on the cement surface in a 24-well plate. The L929 cells were subcultured using α-MEM supplemented with 10% horse bovine serum and incubated at 37 °C under 5% CO_2_. The L929 cells of the tenth passage were detached using 0.25% trypsin in phosphate-buffered saline and resuspended in α-MEM until experimental use. Cell proliferation was investigated using the WST-8 [2-(2-methoxy-4-nitrophenyl)-3-(4-nitrophenyl)-5-(2,4-disulfophenyl)-2*H*-tetrazolium, monosodium salt] assay. WST-8 is reduced by dehydrogenases in cells to yield a yellow product (formazan), which is soluble in the tissue culture medium, and the amount of formazan generated by dehydrogenase activity in cells is directly proportional to the number of living cells. The OD450 values of the WST-8-treated medium (10% *v*/*v*) was determined after a 2 h incubation by using an enzyme-linked immunosorbent assay reader.

### 2.7. Radiopacity

The cement samples with BS were fabricated as described previously but supplemented with an additional 10 wt % BS (98%, Alfa Aesar, Ward Hill, MA, USA). The X-ray opacity measurements of 3-mm-thick samples of CPC/BS, CPC/H2/BS, CS/H2/BS, CPC/H2/CS5/BS, CPC/H2/CS10/BS, CPC/H2/CS5Z/BS, and CPC/H2/CS10Z/BS were recorded. The X-ray generator (Shimadzu CIRCLEX 1/2P33D-85, Shimadzu, Kyoto, Japan) at National Yang-Ming University was used to irradiate the pure CPC and CPC/BS samples (*n* = 6). The exposure parameters were 42 kVp and 200 mAs. The OD values were recorded and calculated using Mephysto software (version 6.30, PTW-New York Co., Hicksville, NY, USA). The radiopacity of the samples was expressed as the thickness equal to that of aluminum by using a pure aluminum step wedge, having a thickness of 0.5–9 mm with equally placed steps of 0.5 mm.

### 2.8. Statistical Analysis

All tests were performed in duplicate to confirm and at least three samples were tested in each study. We used Origin software (Origin 8.0, Microcal Software Inc., Northampton, MA, USA) for statistical analysis. Data were analyzed through one-way analysis of variance, followed by the Bonferroni tests for post hoc comparisons; *p* < 0.05 was considered statistically significant.

## 3. Results

**Figure 1:**
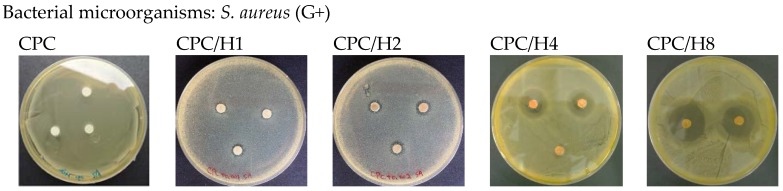
Inhibition zone (*S. aureus*) of CPC, CPC contained 0.001 g hinokitiol (CPC/H1), CPC contained 0.002 g hinokitiol (CPC/H2), CPC contained 0.004 g hinokitiol (CPC/H4), and CPC contained 0.008 g hinokitiol (CPC/H8). The data in the same statistical level was marked with the same letter (a, b, c, d, and e) after the data.

**Figure 2:**
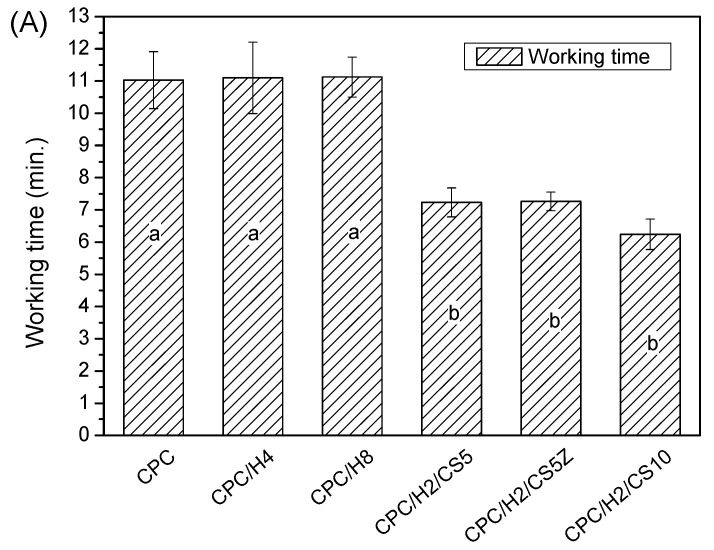

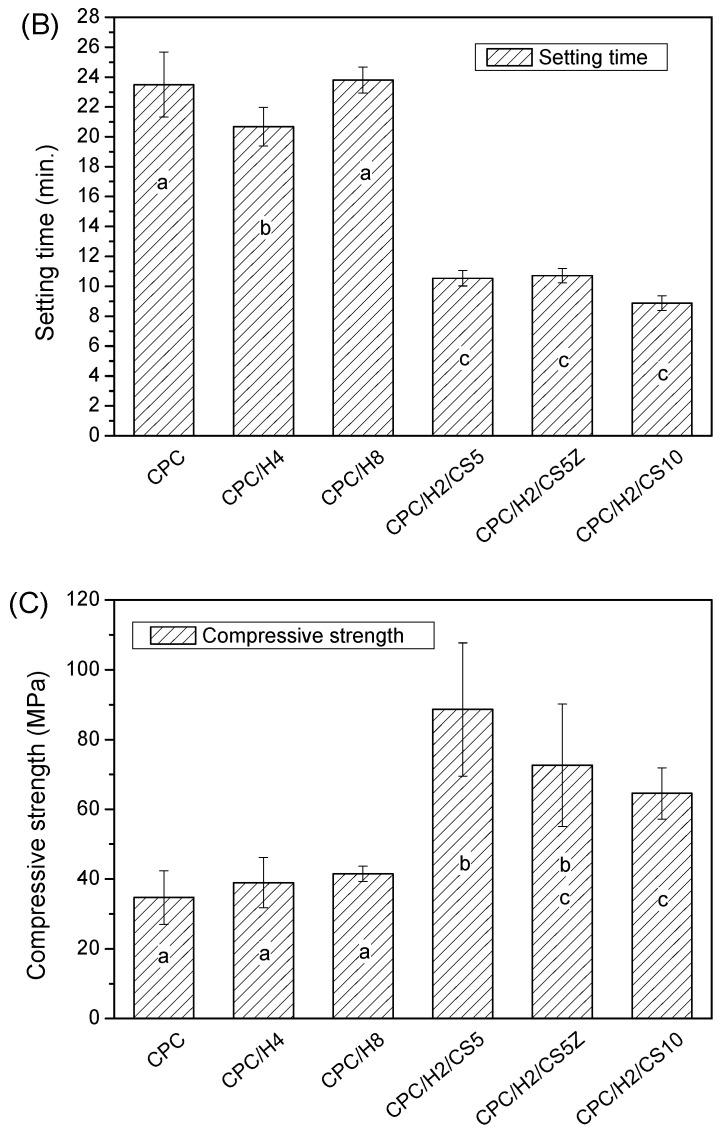
The working time (**A**); setting time (**B**); and compressive strength (**C**) of CPC; CPC/H4, CPC/H8, CPC/H2/CS5, CPC/H2/CS5Z, and CPC/H2/CS10. The data in the same statistical level was marked with the same letter (a, b, and c) on the bar.

**Figure 3:**
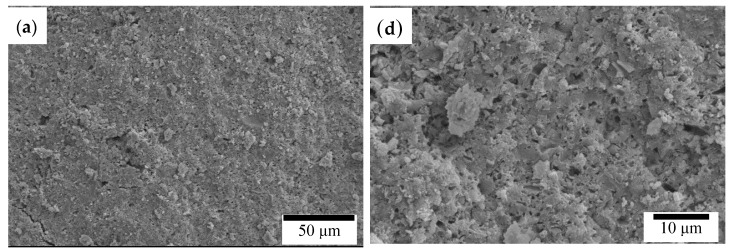

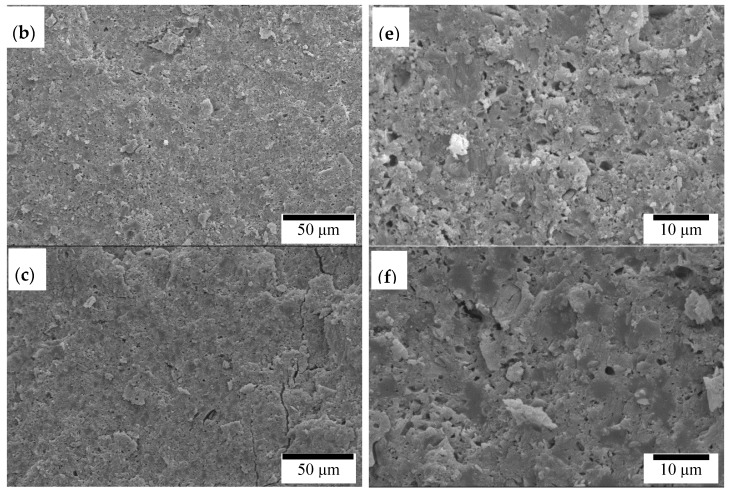
SEM observation of CPC (**a**) 500× (**d**) 2000×; CPC/H4 (**b**) 500× (**e**) 2000×; and CPC/H8 (**c**) 500× (**f**) 2000×.

**Figure 4:**
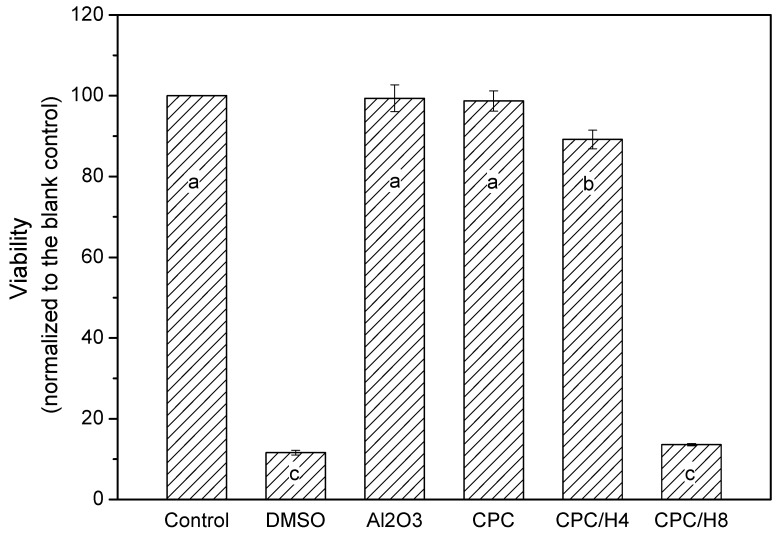
Cytotoxicity test (24 h) of CPC, CPC/H4 and CPC/H8 compare to the positive control (10% DMSO) and blank control (Al_2_O_3_). The data in the same statistical level was marked with the same letter (a, b, and c) on the bar.

The pure (100%) CPC sample did not exhibit antibacterial activity against *S. aureus*. However, when hinokitiol was added to the CPC, a dose-dependent inhibitory effect was observed on *S. aureus* (Figure 1), and the inhibition zone increased with the amounts of hinokitiol in the CPC significantly. The working time, setting times and compressive strength of the CPC were not altered by hinokitiol addition, even at the highest hinokitiol dosage of 0.008 g (in CPC/H8; Figure 2). However, the addition of CS obvious reduced the working time and setting time of CPC/H2 cement. The working time of CPC/H2/CS5, CPC/H2/CS5Z, and CPC/H2/CS10 (6–7 min) were much lower than that of CPC and CPC with hinokitiol (10–12 min). Notably, the addition of CS largely increased the compressive strength of CPC. The compressive strength of CPC/H2/CS5 (88.6 ± 19.1 MPa), CPC/H2/CS5Z (72.6 ± 17.5 MPa), and CPC/H2/CS10 (64.6 ± 7.4 MPa) were significant higher than that of CPC (34.0 ± 7.9 MPa), CPC/H4 (38 ± 6.9 MPa) and CPC/H8 (41.5 ± 2.3 MPa) (Figure 2C). The SEM observations (Figure 3) confirmed that the cements reacted after immersion in water at 37 °C for 24 h, and the particles were connected through apatite formation. Notably, CPC/H8 appeared to be highly homogenous; therefore, morphological analysis revealed that CPC/H8 was denser than CPCs without hinokitiol (enlarged picture, Figure 3f). The cytotoxicity test (Figure 4) results revealed that the CPC supplemented with less than 0.004 g of hinokitiol per 0.8 g of CPC had acceptable biocompatibility. The viability of cells in CPC/H8 was 70% lower than that in the control group, indicating that a high dosage of hinokitiol exhibits cytotoxicity. Therefore, the CPC was supplemented with only 0.001 and 0.002 g of hinokitiol (H1 and H2, respectively) per 0.8 g CPC for the subsequent evaluations.

**Figure 5:**
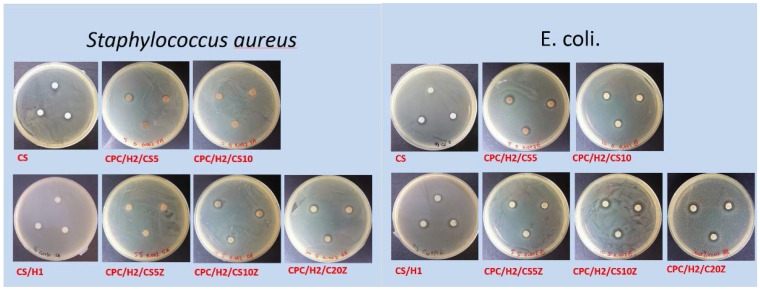
Summarized of the diameter of inhibition zones (in mm) of CS, CS/H1 and CPC/H2 with different CS compositions on *S. aureus* and *E. coli*. The data in the same statistical level was marked with the same letter (a, b, c, and d) after the data.

After determining the effects of different hinokitiol proportions on the basic properties of the hinokitiol-containing CPCs, we examined the effects of pure CS and CS/Z on CPC/H2. Figure 5 presents a summary of the antibacterial activities of different cement compositions. As expected, CS exerted strong inhibitory effects on *S. aureus* and *E. coli*; CS and 0.001 g of hinokitiol exerted a synergetic antibacterial effect on bacteria, and the inhibition zones of CS/H1 were higher than those of pure CS. The inhibition zone of CPC/H2 for *S. aureus* was 2.95 ± 0.54 mm (Figure 1). CPC/H2 with 5 wt % CS (CPC/H2/CS5) had a slightly increased inhibitory effect, with inhibition zones of 3.11 ± 0.69 and 3.64 ± 0.25 mm for *S. aureus* and *E. coli*, respectively. However, an increase in CS amount to 10% (CS10) in CPC/H2 resulted in the reduction of inhibitory ability. The inhibition zones of CPC/H2/CS10 for *S. aureus* and *E. coli* were 0.75 ± 0.25 and 2.45 ± 0.25 mm, respectively, which are lower than those of CPC/H2/CS5. When 5, 10, and 20 wt % CS/Z were added to CPC/H2, the inhibition zones for *S. aureus* were 0.50 ± 0.29, 4.02 ± 0.99, and 5.03 ± 0.27 mm, respectively. However, CPC/H2/CSZ was more effective in *E. coli* growth inhibition; the inhibition zones of CPC/H2/CS5Z, CPC/H2/CS10Z, and CPC/H2/CS20Z were 3.87 ± 0.25, 6.08 ± 1.44, and 5.29 ± 0.25 mm, respectively. The antibacterial activities of CPC/H2/CS5Z and CPC/H2/CS10Z were higher than those of CPC/H2/CS5 and CPC/H2/CS10.

**Figure 6:**
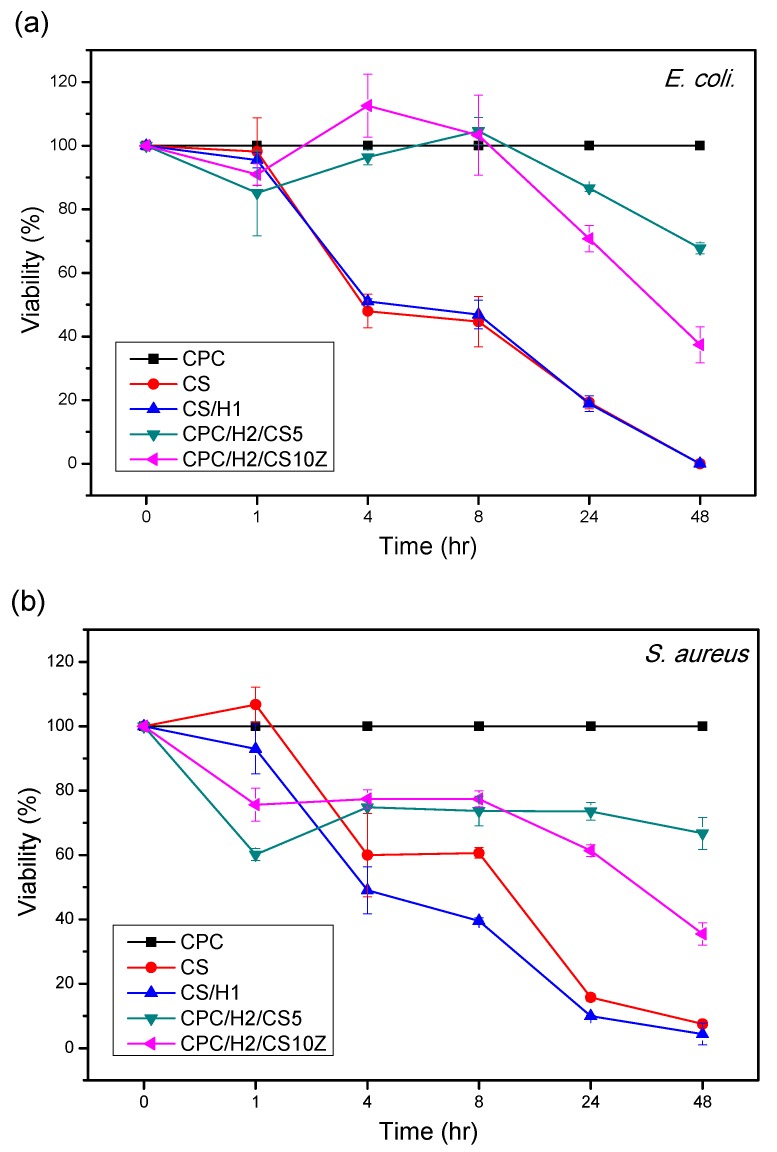
Relative viability of *S. aureus* (**a**) and *E. coli* (**b**), cultured in PBS for up to 48 h. The time profiles of bacteria viabilities indicate the inhibition ability of CS, CS/H1, CPC/H2/CS5, and CPC/H2/CS10Z (optical density percentages at 600 nm normalized to CPC as 100%).

As indicated in Figure 6, the viabilities of *S. aureus* and *E. coli* decreased with increasing incubation time when cultured with pure CS, CS/H1, CPC/H2/CS5, and CPC/H2/CS10Z. Pure CS and CS/H1 possessed higher inhibitory ability than CPC/H2/CS5 and CPC/H2/CS10Z, where *S. aureus* and *E. coli* cells were completely killed after 48 h. The bacteria cultured with CPC/H2/CS5 and CPC/H2/CS10Z were also inhibited, with viabilities of approximately 65% and 40% of those of the control group, respectively. CPC/H2/CS5 and CPC/H2/CS10Z exhibited higher antibacterial activities against *E. coli* than against *S. aureus* at 1, 4, and 8 h after incubation. Notably, the CPC samples with H2 (CPC/H2/CS5 and CPC/H2/CS10Z) exerted early inhibitory effects than those with CS and CS/H1; that is, cell viability was lower in CPC/H2/CS5 and CPC/H2/CS10Z than those in CS and CS/H1 at 1 h after incubation. After a 4–24 h incubation, CPC/H2/CS5 and CPC/H2/CS10Z had low inhibitory abilities. By contrast, the inhibitory effects of CS and CS/H1 were more linearly correlated with the incubation time.

**Figure 7:**
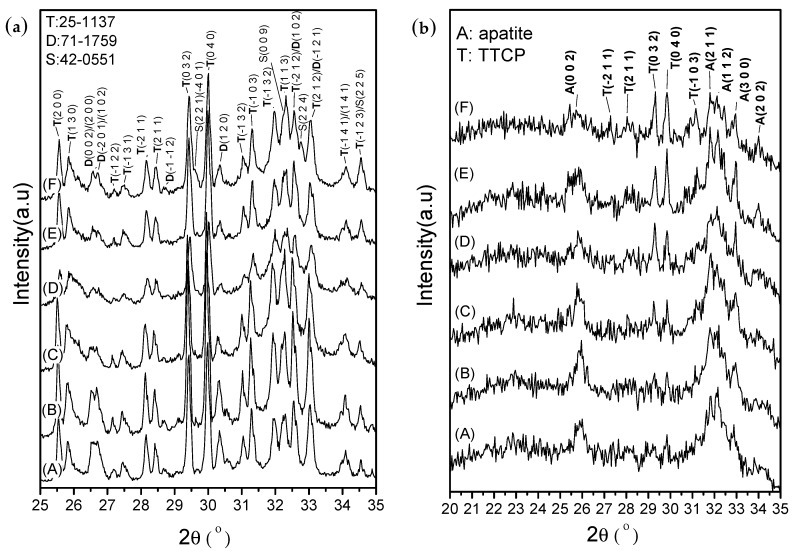
X-ray diffraction patterns of samples after setting for 1 h (**a**) and for 24 h (**b**). (A) CPC; (B) CPC/H2; (C) CPC/H2/CS5; (D) CPC/H2/CS5Z; (E) CPC/H2/CS10; (F) CPC/H2/CS10Z.

After setting for 1 h, the XRD patterns (Figure 7a) revealed that the phase structures remained mostly similar to the original CPC powder obtained after mixing TTCP (JCPDS 25-1137) and DCPA (JCPDS 71-1759). The CS phase was not observed in the diffraction patterns of CPC/H2/CS5, CPC/H2/CS5Z, and CPC/H2/CS10 (Figure 7a, lines C, D, and E, respectively). However, the CS (JCPDS #42-0551) phase was clearly observed in the diffraction pattern of CPC/H2/CS10Z. The diffraction peaks at 29.55°, 32.27°, 32.76°, and 34.55° in CPC/H2/CS10Z were assigned to the (2 2 1)/(−4 0 1), (0 0 9), (2 2 4), and (2 2 5) planes of CS (JCPDS #42-0551), respectively. The setting reactions of the pure CPC and CPC/H2 samples were completed after 24 h (Figure 7b), in which most phases were converted to the apatite phase. The diffraction peak at approximately 26° and broad peaks at 31°–33° corresponded to the typical apatite peaks of the (0 0 2), (2 1 1), (1 1 2), and (3 0 0) planes. However, CPC/H2/CS5, CPC/H2/CS5Z, CPC/H2/CS10, and CPC/H2/CS10Z showed retarded apatite conversion; the peaks corresponding to the apatite phase decreased, and the concentration of unreacted TTCP increased with increasing CS content.

**Figure 8:**
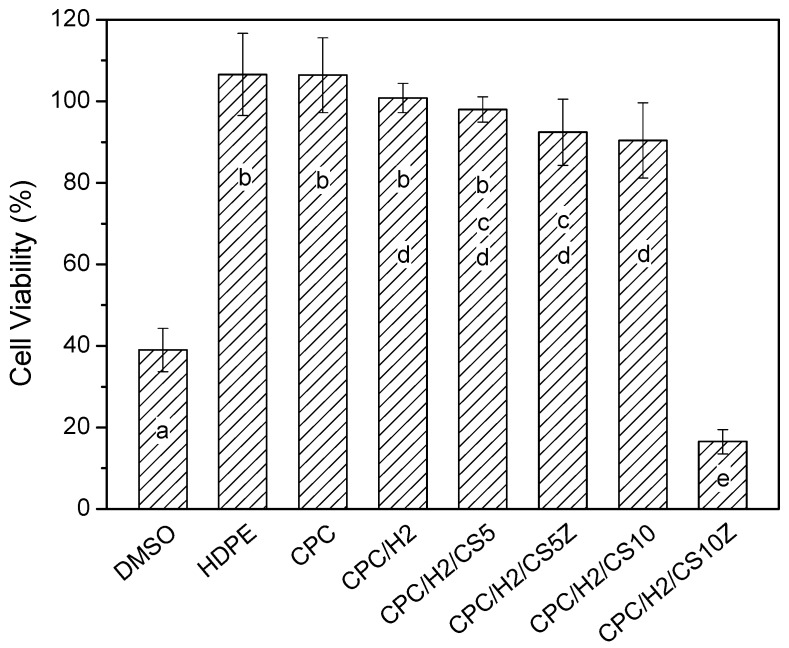
The relative cell viabilities (%) of CPC, CPC/H2, CPC/H2/CS5, CPC/H2/CS5Z, CPC/H2/CS10 and CPC/H2/CS10Z compare with HDPE (positive control group), DMSO (negative control group), and a blank group (as 100% cell viability) obtained in 24 h cultures. The data in the same statistical level was marked with the same letter (a, b, c, d, and e) on the bar.

**Figure 9:**
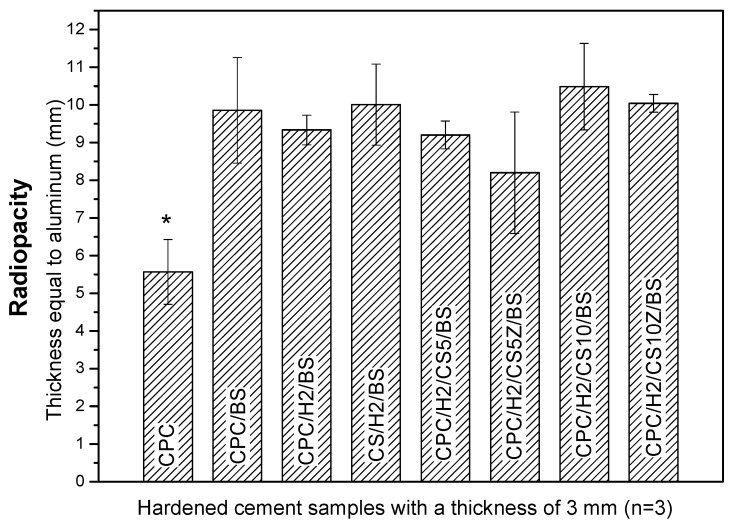
Radiopacity of CPC, CPC/BS, CPC/H2/BS, CS/H2/BS, CPC/H2/CS5/BS, CPC/H2/CS5Z/BS, CPC/H2/CS10/BS, CPC/H2/CS10Z/BS. The radiopacity of samples were expressed as thickness equal to aluminum. The asterisk means the *p* value is below 0.001 when compared with other groups.

The CPCs possessed superior cytocompatibility, and the viability of cultured cells in the pure CPC sample was the same as that in the high-density polyethylene (HDPE) positive control (Figure 8). In particular, the cell viabilities in CPC/H2, CPC/H2/CS5, CPC/H2/CS5Z, and CPC/H2/CS10 were identical and were more than 70% of the blank control group. The cell viabilities in CPC/H2 and CPC/H2/CS were statistically equivalent to those in the pure CPC and the HDPE positive control. However, the cell viability in CPC/H2/CS10 was very low (less than 20% of the blank control group), and it was as toxic as the 10% dimethyl sulfoxide negative control. The radiopacity of the CPCs was increased considerably by the addition of 10 wt % BS in each group (Supplements; Figure S1). The OD value of the 3 mm pure CPC was almost equal to that of 5.5 mm pure aluminum (Figure 9). BS improved the radiopacity of all groups; the OD values of the BS-containing CPCs were equal to those of 8–10 mm pure aluminum. Moreover, CS and CS/Z did not considerably alter the radiopacity of the CPCs.

## 4. Discussion

The findings of this study confirm that appropriate amounts of hinokitiol (a cypress extraction) can be used in CPCs for antimicrobial applications without altering their working or setting time, mechanical properties, or cytocompatibility. A hinokitiol concentration of approximately 0.25–0.5 wt % of the amount of the CPC powder (CPC/H2 or CPC/H4) endowed appreciable antimicrobial properties without exerting cytotoxic effects or altering handling or mechanical properties. Hinokitiol is insoluble in aqueous systems; therefore, it may not participate in the apatite formation reaction in CPCs. Our supplemental XRD and Fourier transform infrared spectroscopy data (Figures S2 and S3) confirmed that the 24 h apatite formation was not altered by the addition of 0.008 g of hinokitiol (in CPC/H8). Notably, the compressive strength of CPC/H8 was slightly higher than those of the other CPC groups (Figure 2), and this may be because hinokitiol can react with calcium ions and form metal complexes [31], as a result, the fracture surface of CPC/H8 revealed a lower porosity level than those of pure CPC and CPC/H4 (Figure 3f). Less porous cements may be advantageous for endodontic applications, and the sealing ability is an important consideration. In a previous study [31], they compared the antimicrobial activity of various hinokitiol complexes with hinokitiol, and their results showed that complex with the dimeric molecular structure such as [Ag(hino)]_2_ would possess higher antimicrobial activity than other types of complex. However, they only compared the antimicrobial activity of pure compound in ppm level. In our study, the antimicrobial activity increased dependently with hinokitiol increment (0.125–1 wt %), as a result CPC/H2 has a significant zone of inhibition, and CPC/H8 has a highest inhibitory effects on *S. aureus*. It is proposed that the Ca complex may form in the present CPC-hinokitiol system, but the free hinokitiol also increased by the increment of hinokitiol. Thus, the antibacterial effects of CPC with hinokitiol is dose dependent. When take biocompatibility into account, the CPC-hinokitiol system containing hinokitiol-calcium complex may present lower toxicity when compared to the hinokitiol-silver complex. Our results demonstrate that CPC incorporated with hinokitiol possessed superior antimicrobial properties. However, high hinokitiol dosages (CPC/H8, 1 wt %) induce cytotoxicity in cements.

MTAs have been used as endodontic materials for numerous years. CS is the main component of commercial MTAs; hence, when CS is mixed with water, it reacts to form calcium silicate hydrate that increases the comprehensive strength of materials, according to the following equation:

Ca_3_SiO_5_ + zH_2_O → Ca_x_Si(OH)_y_·nH_2_O + (3 − x) Ca(OH)_2_

Our XRD results show that CS had obstructive effects on apatite formation in CPCs, which is consistent with the finding of a previous study [32]. In the same paper, they also addressed the fact that the incorporation of CS to CPC would come with both the slight increase of mechanical strength and the reduction of setting time (18–30 min), but the effect of CS is not significant without addition of hardener (Na_2_HPO_4_). Our results demonstrated that the addition of CS shortened the working time and setting time of CPC/H2 significantly, which meant that in clinical applications the cement would be easy to manipulate and would be hardened within an appropriate time (10–12 min). As a comparison, the setting time of MTA was over several hours to days, which has been criticized for treating complex case such as retrograde filling [13,15]. On the other hand, the present CPC incorporate of CS has a dramatically strengthening effect. The mean compressive strength of CPC/H2/CS5 is 88.6 MPa, which is double of CPC and CPC with hinokitiol. However, the compressive strength of CPC/H2/CS10 (10 wt % CS) was lower than CPC/H2/CS5. It is speculated that the present of 5 wt % CS may retard the apatite formation of CPC, but at the same time the hydrated CS forming CSH would possible bind the apatite and residual calcium phosphates particles thus increase the strength. In addition, the 10 wt % CS become a serious impediment to apatite formation of CPC, and the low-strength CSH would become a continuous fracture path as a result the strength was decreased. Higher strength and lower setting time can be a benefit for sealing the defect, condensation and built-up in several difficult filling cases. However the indication has not been specified and the clinical feasibility should await more evaluation. Another study also reported the same tendency in an α-Ca_3_(PO_4_)_2_-based CPC, that the compressive strength of the α-Ca_3_(PO_4_)_2_-based CPC increased after the addition of 5 wt % CS, [33]. By contrast, the compressive strength of the α-Ca_3_(PO_4_)_2_-based CPC containing 10 wt % CS decreased drastically due to the delayed hydrolysis of α-Ca_3_(PO_4_)_2_. However, the mentioned studies have not investigated the antibacterial properties of CS-containing CPCs, which is a main requirement of endodontic materials.

In this study, we found that CS and hinokitiol exerted a synergic antibacterial effect (Figure 5). CPC/H2/CS5 had comparable antibacterial properties to the CS group. Their inhibitory effect is significant and the same regardless of *S. aureus* or *E. coli*. However, the effects were not dose-dependent. CPC/H2/CS10 had a lower inhibitory effect (than CPC/H2/CS5), against *S. aureus* and *E. coli* respectively. This is possibly because the active antibacterial ingredient, CaOH (CS hydrolysis product), was neutralized by phosphoric acid, a DCPA hydrolysis product. Thus, the cement-to-apatite transformation was suspected been stopped by the exhaustion of acid. These findings could be confirmed by the changes in the phase structures of the CS-containing CPCs (CPC/H2/CS5, CPC/H2/CS5Z, CPC/H2/CS10, and CPC/H2/CS10Z) after 24 h. The unreacted TTCP concentration increased with increasing CS content; however, DCPA was completely dissolved after 24 h (Figure 7b). Clearly, the CPC apatite formation was retarded by CS increment.

CPCs with 5, 10, and 20 wt % CS/Z showed a linear correlation with antibacterial properties. However, the post-hoc comparison shown no differences between CPC/H2/CS10Z and CPC/H2/CS20Z, regardless of *S. aureus* or *E. coli*. The effects of ZnO doping on antibacterial properties were significant when the CS ratio was higher than 10 wt %. The Bonferroni test results demonstrated that inhibition zones of CPC/H2/CS10Z were higher than CPC/H2/CS10, regardless of *S. aureus* or *E. coli*. The addition of 5 wt % ZnO in CS stabilized the CS phase by replacing the O–Ca–O bond with a stronger O–Zn–O bond [34]. The XRD patterns of CPC/H2/CS10 and CPC/H2/CS10Z (Figure 7a) revealed that the CS phase presented distinguished diffraction peaks only in CPC/H2/CS10Z. CPC/H2/CS10Z had a higher inhibitory ability than CPC/H2/CS10, indicating that under conditions of higher CS content, the zinc ions may enhance antibacterial effects. A study reported that hinokitiol can form metal complexes such as Zn(hinokitiol)_2_ or other diketonates L to increase antibacterial activity [22]. The bacterial viabilities decreased with increasing incubation time (Figure 6). However, the inhibitory mechanism of CPC/H2/CS5 and CPC/H2/CS10Z is likely different from that of CS and CS/H1; the bacterial viabilities in CS decreased linearly with incubation time, and those in CPC/H2/CS5 and CPC/H2/CS10Z showed a two-step decreasing curve. This discrepancy could be because of the unique paradoxical inhibition phenomenon of hinokitiol, where the inhibition can occur at both low and high dosages [35]. The hydrophobic hinokitiol was released quickly in the early stage, as a result the inhibitory effects of CPC/H2/CS5 or CPC/H2/CS10Z were strong after 1 h and remained steady in the 2–8 h time interval. At 24 and 48 h, the low-concentration hinokitiol still has inhibitory ability, the bacterial viabilities of cells incubate with CPC/H2/CS5 and CPC/H2/CS10Z decreased again and the decreasing slope is the same as that of the CS group. This phenomenon has not been reported in other hinokitiol-containing calcium silicate cement systems [23]. The antimicrobial activity of their calcium silicate cement is contributed from Ca(OH)_2_, which is the hydrolysis product of main componentsβ-Ca_2_SiO_4_ (or calcium silicate hydrate). The β-Ca_2_SiO_4_ cement already has good inhibitory effects against *Enterococcus faecalis* without addition of hinokitiol, while the additional hinokitiol (concentrations from 0.01 to 10 mM) provide only little effects to increase the antimicrobial activity of the hinokitiol-modified CS cements [23]. Our results demonstrated that when 0.25% hinokitiol is added to CPC with 5–10% CS (Ca_3_SiO_5_) they provide similar inhibitory effects to that of pure CS (Ca_3_SiO_5_), and greatly improve the antibacterial effect of CPC. Moreover, low solubility can maintain the sealability thus is a highly demanded property for materials in use as an endo filler. The phase of calcium silicate cement after setting consists of mainly β-dicalcium silicate (β-Ca_2_SiO_4_), calcium silicate hydrate, and calcium carbonate [23], which all possess higher solubility [25,26,27,28,29] than apatite [Ca_10_(PO_4_)_6_(OH)_2_], the final product of the present CPC system. On the other hand, the diametral tensile strength (6 mm diameter × 3 mm thickness) of CS (represent of β-Ca_2_SiO_4_ in [23]) with hinokitiol (2.39–2.65 MPa) is much lower than the present compressive strength (6 mm diameter × 12 mm height) of CPC-hinokitiol system (35–40 MPa). The inherited osteogeneses potential of CPC makes it possible for other hard tissue repair applications that might not be feasible for calcium silicate cements. Given that the load-bearing medical application is the main drawback of calcium silicate cements, calcium silicate cements didn’t show cytotoxicity in previous studies [23,34,36,37,38].

Recently, many studies have confirmed the biomimetic effects of Si and the enhanced osteoconductivity of endodontic materials; silicates provide acceptable biocompatibility, enhance cell proliferation, and activate bone-related gene expression [36,37,38,39,40]. However, some studies have reported that the antimicrobial properties of calcium silicate-based endodontic filling materials are always associated with a potential increase in cytotoxicity [41,42,43]. The present in vitro results show that although CS and CS/H exerted strong antibacterial effects, their cytotoxicity was higher than that of pure CPCs. CPC/H2/CS10Z exerted the highest antibacterial effects, but increased cytotoxicity would be deleterious to its compatibility. Radiopacity is an important characteristic of endodontic materials, which enables a strong contrast between endodontic materials and tooth structures/tissues, thus improves the traceability, sensitivity and correctness of medical imaging diagnosis [44]. In our previous studies, CPCs containing 10 wt % BS had improved radiopacity and a lower rate of blood coagulation, which conferred increased antiwash features to the setting CPCs. This might be an advantage for use as endodontic materials, but of course further confirmation of the seal ability is needed. In the present study, 10 wt % BS improved the radiopacity of all CPC groups without altering their handling or mechanical properties.

## 5. Conclusions

CPCs doped with 0.25–0.5 wt % hinokitiol had appreciable antimicrobial properties without alterations in their working or setting times, handling or mechanical properties, or cytocompatibility. The incorporation of CS shortened the working time and setting and largely improved the compressive strength of CPC/H2 cement. CS and hinokitiol exerted a synergic antibacterial effect, CPC/H2/CS5 had comparable antibacterial properties to the CS group, and CPC/H2/CS10Z had a higher antibacterial activity level than pure CS. The addition of 10 wt % BS doubled the CPC radiopacity. The findings of the present study demonstrate that hinokitiol and CS can improve the antibacterial properties of CPCs, and they can thus be considered for endodontic applications.

## Figures and Tables

**Table:** 

Sample Code	Powder (g) (TTCP/DCPA)	Liquid (mL) (NH_4_)_2_HPO_4_	Hinokitiol (g)/ (% in Powder)	Diameter of Inhibition Zone
CPC	0.8	0.28	0/(0)	0 mm ^a^
CPC/H1	0.8	0.28	0.001/(0.125)	1.55 ± 0.49 mm ^b^
CPC/H2	0.8	0.28	0.002/(0.25)	2.95 ± 0.54 mm ^c^
CPC/H4	0.8	0.28	0.004/(0.5)	14.70 ± 0.40 mm ^d^
CPC/H8	0.8	0.28	0.008/(1.0)	21.51 ± 0.33 mm ^e^

**Table:** 

Sample Code	CPC %	Hinokitiol % in Powder	CS %	ZnO % in CS	Diameter of Inhibition Zone	
					*S. aureus* (G+)	*E. coli*. (G−)
CS	0	0	100	0	4 ± 0.45 ^a^	4 ± 0.5 ^a,c,d^
CS/H1	0	0.125	100	0	6 ± 0.33 ^b^	7 ± 0.3 ^b–d^
CPC/H2/CS5	95	0.25	5	0	3.11 ± 0.69 ^a^	3.64 ± 0.25 ^a,d^
CPC/H2/CS5Z	95	0.25	5	5	0.50 ± 0.29 ^c^	3.87 ± 0.25 ^a,c,d^
CPC/H2/CS10	90	0.25	10	0	0.75 ± 0.25 ^c^	2.45 ± 0.25 ^a^
CPC/H2/CS10Z	90	0.25	10	5	4.02 ± 0.99 ^a^	6.08 ± 1.44 ^b–d^
CPC/H2/CS20Z	80	0.25	20	5	5.03 ± 0.27 ^a,b^	5.29 ± 0.25 ^d^

## Supplementary Materials

The following are available online at www.mdpi.com/1996-1944/10/11/1256/s1, Figure S1: X-ray photo for determine the radiopacity of CPC with H2/CS and with 10% BS. Figure S2: XRD diffraction patterns of CPC, CPC/H4, and CPC/H8 after setting for 24 h with compare to hinokitiol original powder. Figure S3: The characteristic FTIR bands of CPC, CPC/H8, and pure hinikitiol.
