# Supplementary material for: Calcium Phosphate Cement with Antimicrobial Properties and Radiopacity as an Endodontic Material
Source: Materials (Basel). 2017 Oct 31;10(11):1256. doi: 10.3390/ma10111256 (PMC5706203; doi:10.3390/ma10111256)
Supplement: Supplementary file 1 [file materials-10-01256-s001.pdf]

Supplementary Materials:

Calcium Phosphate Cement with Antimicrobial Properties and Radiopacity as an Endodontic Material

Tzong-Ming Shieh [1,2], Shih-Ming Hsu [3], Kai-Chi Chang [4,5], Wen-Cheng Chen [1,4,5,†] and Dan-Jae Lin [1,2,*]

Figure S1 provide the raw data of radiopacity test and shows that the radiopacity of the CPCs was increased considerably by the addition of 10 wt% BS in each group.

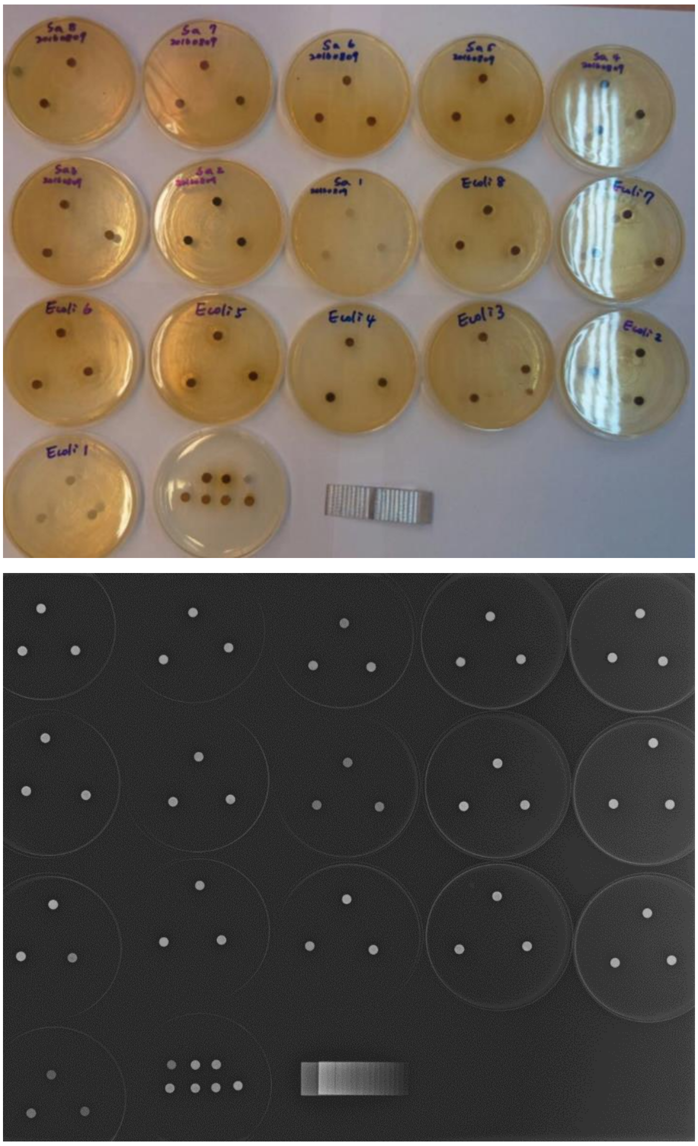

Figure S1. X-ray photo for determine the radiopacity of CPC with H2/CS and with 10% BS. The rectangle block at the bottom is the stepped pure aluminum for calibration.

The XRD pattern (Fig. S2) revealed that pure hinokitiol has crystalline structure, however, it did not alter the apatite conversion of cement reaction at these low dosages. The CPC/H4 and CPC/H8 have the same phase structure as pure CPC. Moreover, the FTIR spectrums (Fig. S3)

demonstrated that additional C=C of aromatic ring and C-H bond due to the doping of hinokitiol was apparently revealed in the CPC/H8.

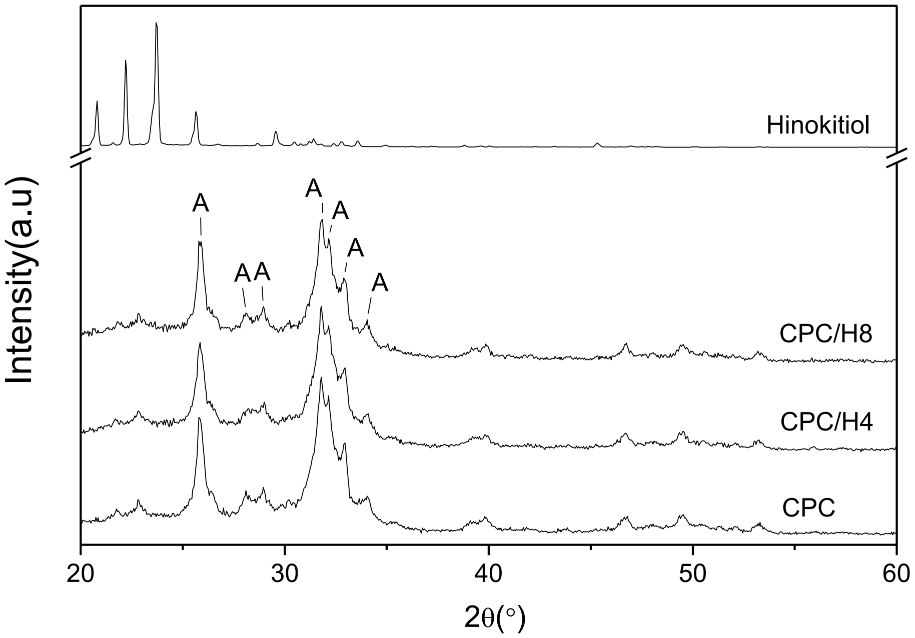

Figure S2. XRD diffraction patterns of CPC, CPC/H4, and CPC/H8 after setting for 24 h with compare to hinokitiol original powder.

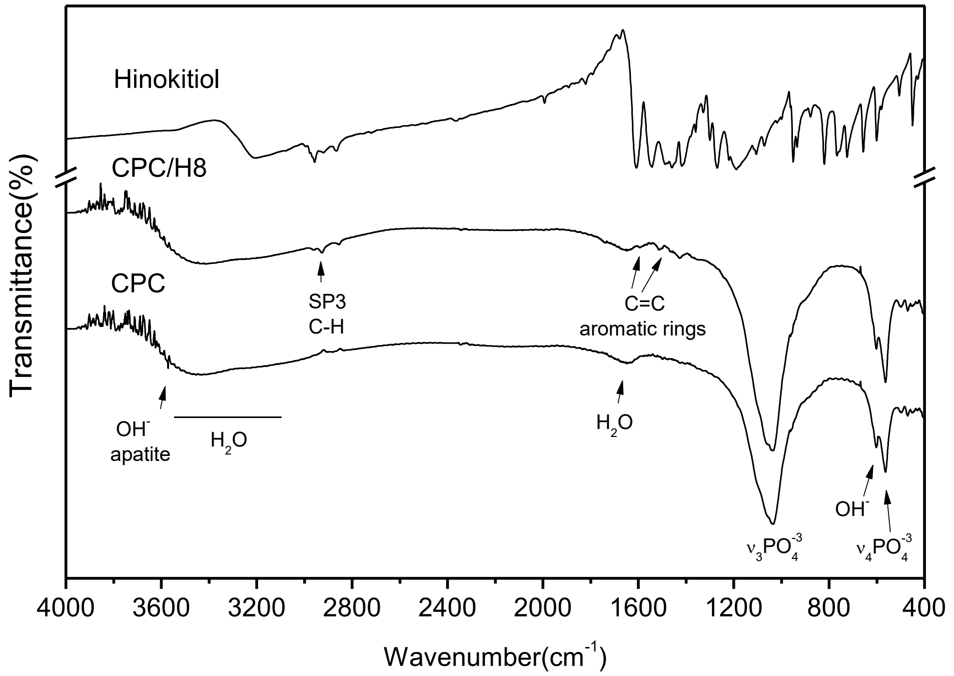

Figure S3. The characteristic FTIR bands of CPC, CPC/H8, and pure hinikitiol. The hinikitiol were obtained from the original powder. The CPC samples were immersed in 37 °C water for 24 h after setting. All the samples for FTIR evaluation were prepared in the 1:100 ratio to KBr and spectrums were measured as the relative percent transmittance.
